# Navigating Non-interventional Post-authorization Studies in East Asia: Regulatory Challenges, Opportunities, and Future Directions

**DOI:** 10.1007/s40264-025-01621-x

**Published:** 2025-10-17

**Authors:** Jami Peters, Ayad K. Ali, Maria Moitinho de Almeida, Keiko Asao, Tarek A. Hammad, Xintong He, Alexander Michel, Annalisa Rubino, Sono Sawada, Rachel E. Sobel, Stefan de Vogel

**Affiliations:** 1 Gilead Sciences Inc., Foster City, CA USA; 2 BeOne Medicines Ltd., San Carlos, CA USA; 3 GSK, Wavre, Belgium; 4 Kappa Medical K.K., Tokyo, Japan; 5 Takeda Development Center Americas, Inc., Cambridge, MA USA; 6 IQVIA, Shanghai, China; 7 Bayer Consumer Care AG, Basel, Switzerland; 8 Gilead Sciences Europe Ltd, Uxbridge, UK; 9 Nippon Boehringer Ingelheim Co., Ltd., Tokyo, Japan; 10 Regeneron Pharmaceuticals, Tarrytown, NY USA; 11 Astellas Pharma Europe B.V., Leiden, The Netherlands

## Abstract

Post-authorization studies (PAS) are often mandated by regulatory authorities as a condition of marketing authorization of pharmaceutical products. This article explores specific regulations and trends in China, Japan, and South Korea, highlighting the scientific and operational limitations that such PAS pose to the stakeholders in these regions including significant variations in regulatory requirements. Pharmacovigilance guidelines and publications on regional regulatory trends were reviewed. Active surveillance studies are widely adopted to fulfill post-authorization requirements in East Asia countries. These are primary data collection studies, i.e., traditional site-based studies that monitor the frequency of all adverse events (and clinical outcomes when requested) of the newly approved pharmaceutical product during a predefined treatment period. Such studies generally present limitations regarding the product’s safety profile characterization, including the absence of a comparator group, selection bias, limited sample size, and considerable resources needed to conduct the studies. These limitations explain the trend toward hypothesis testing studies, conducted with secondary data (e.g., large electronic database studies) as preferred over traditional active surveillance studies. Harmonizing regulatory approaches and enhancing access to comprehensive data sources are critical for generating fit-for-purpose evidence to support regulatory decision making in these regions. Therefore, we propose a decision tool to assist with the planning of PAS in China, Japan, and South Korea. This article is endorsed by the International Society for Pharmacoepidemiology (ISPE).

## Key Points

**Table:** 

To better understand drug risks, the worldwide regulatory landscape is evolving towards post authorization studies targeting specific safety issues.
To optimize the impact of PAS in China, Japan, and South Korea, it is imperative to focus on harmonizing regulatory practices, improving data accessibility, and embracing innovative analytical methods.
As global pharmacovigilance collaboration continues to grow, East Asia has a significant opportunity to lead in establishing a more cohesive and efficient PAS framework, ensuring the safe and effective use of pharmaceutical products worldwide.

## Introduction to Post-Authorization Studies

The purpose of this work is to inform pharmacoepidemiologists designing observational studies in China, South Korea, and Japan on the current regulatory landscape and to encourage continued discussion with regulators on the acceptance of alternatives to active surveillance studies such as hypothesis-testing studies**.** Routine pharmacovigilance forms a key part of the benefit-risk evaluation in China, Japan, and South Korea, where regulatory authorities have robust adverse drug reaction (ADR) reporting systems [3, 21, 28, 35]. The Korean Institute of Drug Safety and Risk Management (KIDS) was established in 2012. The KAERs system was developed by KIDS, which has stored all adverse events (AEs) since 2012. In addition to routine pharmacovigilance from ADR reporting systems, regulatory authorities in China, Japan, and South Korea generally request specific post-authorization studies (PAS) as a condition of the marketing authorization of pharmaceutical products. The submission of a risk management plan (RMP) summarizing the important identified risks, important potential risks, and missing information at the time of marketing authorization may inform the planning of PAS that can fill the knowledge gap about the benefit-risk profile of a product.

Globally, PAS is required by regulators to monitor AEs, fully characterize selected safety concerns, and confirm drug safety and effectiveness in patient populations that were excluded from clinical trials. In Asia, countries including China, Japan, and South Korea have established PAS requirements to confirm drug safety and effectiveness post-approval. These requirements are often due to concerns surrounding the small number of patients included in global registrational trials and possible genetic and environmental factors that may predispose certain populations to differential drug safety effects.

In Japan the re-examination procedure aims to reconfirm the safety and effectiveness of a pharmaceutical product in a defined time window. During the product filing, PAS may be mandated by the regulatory agency for implementation and completion during the re-examination period, with PAS results being submitted at pre-defined intervals during that period.

PAS may be designed as either primary data collection studies or as studies that make secondary use of data collected for purposes other than the study itself (e.g., database studies). Active surveillance studies (i.e., drug use result surveys, all-case surveillance) are usually observational single-arm descriptive studies requiring the systematic collection of all AEs and/or ADRs from the treated population in selected study sites. Additional data may also be required on a case-by-case basis to investigate the effectiveness or other aspects of the treatment regimen (such as hepatic or renal laboratory values). Patients are often enrolled in active surveillance studies through informed consent (though not required for regulatory-mandated PAS in Japan) and are treated with routine clinical care.

While active surveillance studies have been the traditional approach for post-authorization safety monitoring, there is a growing recognition of the value of conducting targeted studies that evaluate specific safety concerns identified in the RMP [11, 13]. Outcomes of interest for targeted studies may be identified via primary data collection or secondary use of databases. With the evolving diversity requirement in clinical trials and robust ADR pharmacovigilance systems, the question remains whether regulatory agencies favor targeted studies that evaluate specific safety concerns versus active surveillance studies that collect data on all AEs (ADRs).

Regulatory agencies in China, Japan, and South Korea have begun to embrace the conduct of targeted hypothesis-testing studies in databases compiling patient-level claims data or medical records. China, Japan, and South Korea have published regulatory guidance on using database studies to characterize the safety profiles of pharmaceutical products, indicating an increasing openness to considering alternative approaches to active surveillance studies.

Harmonizing regulatory approaches and enhancing access to comprehensive data sources are critical for generating fit-for-purpose evidence to support regulatory decision-making in these regions. Therefore, we propose a decision tool to assist with the planning of PAS in China, Japan, and South Korea. China, Japan, and South Korea were selected for this review based on author experience conducting PAS in these regions. Future publications reviewing PAS requirements in other countries in the Asia Pacific region would benefit the pharmacoepidemiology community.

## Regulations for Post-Authorization Studies (PAS) in China, Japan, and South Korea

This section delves into the regulations related to PAS in China, Japan, and South Korea, emphasizing the key similarities and differences across the countries. The guidelines listed below, albeit not exhaustive, have been commonly referenced in communication from regulatory agencies.

### China

- Guidance on Using Real-World Evidence to Support Drug Development and Regulatory Evaluation (2020)
- National Medical Products Administration (NMPA) Good Vigilance Practice (2021)
- Guidance on the Design and Protocol Development of Real-World Studies for Drugs (2023)

The evolving landscape of the pharmacovigilance systems in China has been recently reviewed [38]. Good Vigilance Practice (GVP) guidance released in China in 2021 explicitly states that primary or secondary data studies may be conducted, and that the MAH should suggest the appropriate study design [28]. However, challenges surrounding the availability and accessibility of national databases in China limit the ability to conduct studies based on secondary use of data. Clarification is needed on whether targeted primary data-collection studies that evaluate specific safety concerns or active surveillance studies that collect all AEs/ADRs would be accepted when database studies are not feasible. In some authors’ experiences, active surveillance studies remain the primary approach to PAS in China.

### Japan

- Ministry of Health, Labour and Welfare (MHLW) Ordinance Related to Standards for Conducting Post-Marketing Surveys and Studies on Drugs (Good Post-marketing Study Practice; GPSP) (MHLW Ordinance No. 171, 20 December 2004, revised on 26 October 2017)
- MHLW Notification: Risk Management Plan Guidance (MHLW Notification No. 0411-(1) of the Safety Division, PFSB and No. 0411-(2) of the Evaluation and Licensing Division, PFSB, 11 April 2012)
- PMDA: Guidelines for the Conduct of Pharmacoepidemiological Studies in Drug Safety Assessment with Medical Information Databases (31 March 2014)
- MHLW Notification: Points to Consider for Ensuring Reliability in Post-marketing Database Surveys of Drugs (MHLW Notification No. 0221-(1) of the Pharmaceutical Evaluation Division, PSEHB 21 February 2018)
- MHLW Notification: Procedures for Developing Post-marketing Database Survey Plan (MHLW Notification No. 0314-(4) of the Pharmaceutical Evaluation Division, PSEHB, No. 0314-(4) of the Safety Division, PSEHB, 14 March 2019, revised on 18 July 2024)

Historically, in Japan, PAS relied on active surveillance studies, including a very few studies with a comparative arm, partly due to the lack of large medical information databases and the focus on signal detection for safety. A risk-based monitoring approach and guidance on the development of an RMP were adopted in 2012 [22]. With the increased availability of medical information databases, the GPSP Ordinance was partially amended, and descriptive or hypothesis-testing studies making use of secondary data were first instituted as a study type in 2017 for regulatory-required PAS. After the GPSP amendment, several guidance documents from MHLW and PMDA were released to support the conduct of database studies [31].

It is noteworthy to mention that the GPSP Ordinance also made explicitly clear that it is acceptable to conduct comparative analyses in active surveillance studies (i.e., it is possible to collect data not only from patients exposed to the drug under surveillance but also from patients not treated with such a drug).

A recent study reviewed the RMPs for drugs approved in Japan between 2016 and 2019. The review shows a marked increase in PAS using databases, from 11.5% in 2017 to 19.2% in 2019 [14]. These observations likely reflect the guidance and support from the PMDA and the accessibility of commercially available databases.

### South Korea

- Guideline for Medical Information Database Research (2021)
- Guidelines on Risk Management Plan for Medicines (A guide for civil petitioner) (2024)

In South Korea, there was an amendment to the Pharmaceutical Affairs Act that became effective on 21 February 2025 which eliminated the re-examination system. Post-marketing safety commitments (including PAS) are now integrated entirely into the RMP. The Ministry of Food and Drug Safety (MFDS) usually mandates that the MAHs conduct an active surveillance study as a condition of approval of any new pharmaceutical product. In addition to the monitoring of all AEs and ADRs in the study population, MFDS also requires effectiveness to be evaluated in active surveillance studies, except in certain circumstances where a release from the MFDS is obtained [27]. The MFDS guidance explicitly outlines key study features expected in active surveillance studies. Additionally, the South Korean authority often requires a minimum sample size of 3,000 or 600 patients for active surveillance studies, depending on specific conditions, unless the MAH provides reasonable evidence for a change. While there is no definitive guidance on how to calculate sample size to propose an alternative to 3,000 or 600, the “rule of 3” − based on AEs requiring special monitoring − is generally accepted by the MFDS. Additional considerations may include disease prevalence, the projected number of patients expected to use the drug based on market conditions, and the sample sizes used for other drugs with the same indication. However, these factors may be subject to further discussion and negotiation by the MFDS. Previous articles highlight the limitation of the sample size requirement, which is not based on statistical considerations, but on government policy for mandatory PAS [12, 37].

The MFDS can conduct hypothesis testing to investigate safety signals emerging from the national ADR reporting system, which supports the emphasis of the MFDS on the benefits of comparative analyses based on the use of secondary data. Further, MFDS has released guidance on the conduct of database studies. Still, it is unclear whether database studies or targeted primary data collection studies would be considered an alternative to active surveillance studies for investigating safety or effectiveness outcomes identified in the RMP. In South Korea, national databases such as the Health Insurance Review and Assessment Service (HIRA) are not accessible to the private sector. This restriction is also the case in many other countries across the globe, where MAHs may only be able to access these data to conduct safety studies via partnership through third parties such as universities or other institutions. Such a model may also provide independent analysis for regulatory evaluation. The results of a post-authorization study that evaluated the safety outcomes of interest using the HIRA database were recently published [2]. Additional guidance or mechanisms in South Korea that would support MAH access to HIRA data for safety studies would be welcomed.

## Regulatory Requirements for Active Surveillance Studies

It is noteworthy that in China, Japan, and South Korea requirements for transparency are limited. PAS details are recommended to be posted on Chinadrugtrials.org in China and the Clinical Research Information Service (CRiS) in South Korea. In Japan, there is a registration database named jRCT based on the provisions of the “Clinical Research Act,” but since PAS are conducted based on the Pharmaceutical and Medical Device Act, registration is not mandatory, and it lacks comprehensiveness. The HMA-EMA Catalogues of real-world data sources and studies and ClinicalTrials.gov may also be used by MAHs to voluntarily report PAS (see Table 1).

**Table 1:** Key features of active surveillance studies requested in China, Japan, and South Korea

Study feature	China	Japan	South Korea
Type of surveillance	Active surveillance (drug intensive monitoring)	Active surveillance (drug use-result surveys)	Active surveillance
Scope	Safety *Effectiveness may be required on a case-by-case basis	Safety *Effectiveness may be required on a case-by-case basis	Safety and Effectiveness
Populations	All patients Special populations	All patients Special populations	All patients Special populations
Sample size	Study-dependent, based on scientific and operational considerations and agreed with NMPA	Study-dependent, based on scientific and operational considerations and agreed with PMDA	3,000 or 600 (minimum) unless MAH provides reasonable evidence to justify a smaller sample
Data collection	Often, medical chart abstraction	Often, medical chart abstraction	Often, medical chart abstraction

*MAH* marketing authorization holders, *NMPA* National Medical Products Administration, *PMDA* Pharmaceutical and Medical Devices Agency

*Outlines of the commitment are publicly available in Risk Management Plans through the PMDA website

Not all PAS study results are published, and publications are often missing key information such as data source (electronic medical records, medical chart abstract, etc.) and data analysis (methods used to correct inconsistencies or errors, missing values, impute values, modify raw data, categorize, analyze, and procedures to control sources of bias and their influence on results). Lack of transparency regarding the full study protocols for PAS that are not required to be disclosed to the public impacts the interpretation of published results when key information is missing. It is not possible to track the number observational studies conducted in these regions due to lack of open-access information. Enhancing transparency could foster learnings exchange, reduce duplication of effort, and help identify trends.

## Limitations of Active Surveillance PAS

While well established, active surveillance PAS present several scientific and operational challenges. The main limitation of single-arm active surveillance is the lack of a comparison group to calculate relative risk; thus, it cannot distinguish between events due to primary disease or other therapies, and those due to the drug under surveillance. Single-arm active surveillance studies are descriptive (i.e., hypothesis-generating) and are not able to validate a safety signal. An analytic study is required to validate any potential safety signal (i.e., hypothesis testing). Additionally, active surveillance is prone to selection biases that are hard to mitigate. For instance, larger medical centers are more likely to participate as study sites due to larger resource availability and research-oriented culture. Furthermore, individual patients may self-select the study population through informed consent when required. The number of enrolled patients is generally insufficient to detect rare events and can also provide unstable frequency estimates of known ADRs. This is aggravated by the challenges of longitudinal follow-up in real-world settings. High rates of loss to follow-up can compromise the robustness of the findings, making it difficult to draw reliable conclusions.

In addition, the operational burden of primary data collection in active surveillance studies is substantial, and healthcare providers often face increased workloads, which can impact patient care. In our collective experience, active surveillance studies often use medical chart abstraction for data collection, which is resource intensive and prone to human error, and inconsistent medical terminology may complicate conversions (e.g., unstructured text to categorical data) [1]. This strains resources and may lead to incomplete or inconsistent data collection. Moreover, the logistics of coordinating multiple sites, training staff, and ensuring adherence to study protocols add layers of complexity. These operational difficulties can detract from the efficiency of active surveillance studies and may compromise the scientific validity or interpretability of the results.

## Database Studies as an Alternative to Active Surveillance in China, Japan, and South Korea

All three countries have nationwide insurance coverage for healthcare services, which provides a large volume of valuable data for real-world studies. Database studies use existing data resources such as electronic medical records, insurance claims, registry data, etc. These studies may be descriptive or analytic. There are many references that have described in detail the strengths and limitations of database studies [11, 39]. Here, we provide a high-level summary of both.

The potential benefits of conducting a database study, compared to an active surveillance study, include an increased sample size to investigate rare safety outcomes, the ability to perform comparative analyses, better options to minimize selection bias, and reduced operational burden on investigators and/or MAH. With the analyses of data ready-available as maintained in large electronic databases, it is possible to achieve timely and robust scientific evidence on a drug’s safety profile, which informs the appropriate use of a pharmaceutical product. This may include the frequency of AEs of interest as identified in the RMP, use in special populations, and early recognition of hazards.

However, these databases were created for a purpose outside of the potential study being proposed, which may impact the characteristics of the data. For instance, the data source needs to be evaluated to consider the availability of key data characteristics to address the research questions, such as exposure, outcome, and covariates. The data also need to be assessed for accuracy, completeness, and traceability. Similar to primary data collection studies, longitudinal follow-up time needs to be evaluated to determine whether there is sufficient time to capture the safety or effectiveness outcome of interest in the database. The coding system of the database needs to be well understood. Many of the biases discussed for active surveillance studies also need to be accounted for when designing database studies, such as selection and channeling biases.

A major challenge for MAHs conducting database studies is timely access to these data. Many databases are only available to academia or governments, and appropriate arrangements with those institutions may cause a substantial delay in the execution of analyses. Additionally, the time lag in making the data available for analysis can compromise a timely assessment of specific safety concerns. Reduction in delays in accessing the data could lead to more database studies as an alternative to active surveillance studies. Our recommendation is to reduce the administrative hurdles that may delay data access, while ensuring that patient privacy matters are fully addressed.

Table 2 presents the most widely used databases in China, Japan, and South Korea. Many more databases are described elsewhere, and disease registries may be considered for future studies [18, 20].

**Table 2:** Databases from China, Japan, and South Korea

Country	Name	Data source type	Estimated number of individuals	Access to data
China	HIS-WCH	EHR	> 5 million	Hospital medical records are usually not linked among hospitals; some hospitals may not have an electronic medical records system
China	NHSA	Claims	> 10 million	Mainly covers the public China health insurance; only accessible to academia or government except for publicly available general data
Japan	MID-NET	Hospital-based claims, DPC data, EHR including laboratory test results	> 8 million	Accessible to MAH; requires expert committee review; does not require IRB review if the study purpose is PAS
Japan	JMDC	Insurance-based claims	17 million	Accessible to private companies; does not require IRB review
Japan	MDV	Hospital-based claims, DPC data, partial laboratory test results	44 million	Accessible to private companies; does not require IRB review
Japan	NDB	Nationwide Insurance-based claims	> 100 million	Accessible to MAH; requires expert committee review and IRB review
South Korea	HIRA	Claims	>50 million	Requires IRB approval; database access requires a local principal investigator; data analysis on HIRA is not accessible outside South Korea, often a 1- to 2-year data lag
South Korea	NHIS	Claims	> 50 million	Requires IRB approval; database access requires a local principal investigator. NHIS is not accessible outside South Korea, often a 1- to 2-year data lag

*DPC* Diagnosis Procedure Combination, *EHR* electronic health records, *HIS-WCH* Hospital information system of West China Hospital, *HIRA* Health Insurance Review and Assessment Service, *IRB* Institutional Review Board, *PAS* post-authorization studies, *MID-NET* Medical Information Database Network, *MDV* Medical Data Vision, *NHIS* National Health Insurance Service, *NHSA* Nation Healthcare Security Administration

## Development of a Decision Tool to Assist with the Planning of PAS in China, Japan, and South Korea

**Fig. 1:**
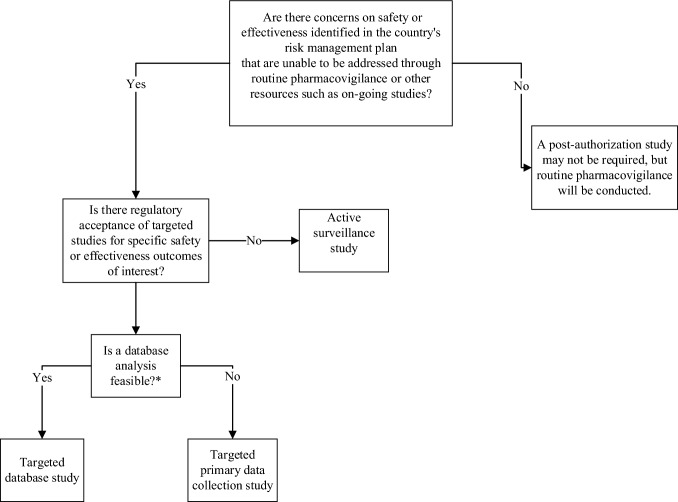
A decision tool to assist in planning for post-authorization studies in China, Japan, and South Korea. *A feasibility analysis is needed to determine whether there are databases that are fit-for-purpose to address the study questions. Some considerations include time to access the database, data quality, population size, study precision, exposures, outcomes, covariates, operations, and accuracy

We developed a decision tool to assist MAHs with initial planning (Fig. 1). This tool provides a high-level overview of study design considerations. In China and South Korea, there is a lack of clarity about the circumstances in which regulatory agencies would consider only requiring routine pharmacovigilance or ongoing studies; in the interim, this option is only applicable to Japan. The success of this workflow also relies on regulatory acceptance of targeted studies as an alternative to active surveillance studies and timely access to databases when proposing a database study. In Japan, the MHLW notification [24] confirms their agreement with this decision process described below.

The next step is to continue open discussion between regulators and members of industry. The hope is that this decision tool may become obsolete because there is clarification on (1) the circumstances in which database studies will be acceptable to a regulatory authority, and (2) how to access different regional datasets.

MAHs, regulatory authorities, and database vendors need to work together to improve the accessibility of databases in these countries to unlock the opportunity to conduct studies using these resources as a scientifically robust alternative to active surveillance studies.

## Conclusion

Post-authorization data on the safety and effectiveness of pharmaceutical products assessed in the real-world setting can confirm the safety profiles in populations not included in the registration trials. However, active surveillance studies face key limitations. To better understand drug risks, the worldwide regulatory landscape is evolving towards PAS targeting specific safety issues. The approach of requiring a large sample size in active surveillance studies to detect rare and uncommon adverse events has shifted to targeted studies, which base sample size calculations on the safety outcomes of interest identified in the RMP [37, 40]. A study is often needed if the PAS aims to validate pre-specified safety or effectiveness concerns outlined in the product’s RMP.

Database studies offer a valid alternative to active surveillance studies, enabling timely and robust pharmacovigilance, and have been well recognized by regulatory authorities for over a decade. The Sentinel Initiative is an established and mature system to monitor the safety of products regulated by the US Food and Drug Administration (FDA) (Center for Drug Evaluation and Research). In the European Union, the Data Analysis and Real-World Interrogation Network (DARWIN EU®) was more recently established to facilitate the generation of real-world evidence to evaluate the safety and effectiveness of pharmaceutical products across Europe.

The secondary use of ready-available health data collected in large electronic databases addresses some of the limitations of traditional active surveillance studies and provides a more comprehensive understanding of a drug’s safety profiles in diverse patient populations.

To optimize the impact of PAS in China, Japan, and South Korea, it is imperative to focus on harmonizing regulatory practices, improving data accessibility, and embracing innovative analytical methods. As global pharmacovigilance collaboration continues to grow, East Asia has a significant opportunity to lead in establishing a more cohesive and efficient PAS framework, ensuring the safe and effective use of pharmaceutical products worldwide. First, legislators and data vendors in China and South Korea should streamline the process for database access for those with long lag times, reducing bureaucratic hurdles and ensuring the timely availability of data for safety analysis. This involves creating clear guidelines and efficient approval processes for accessing these databases. Second, a collaboration between the regulatory agency, MAH, and academic institutions is crucial in enhancing the use of RWD for drug-safety monitoring. Joint initiatives and partnerships can help share best practices and develop innovative methodologies for RWD studies. By improving the accessibility of these data sources and fostering collaboration among stakeholders, we can enhance the efficiency and effectiveness of PAS, ultimately benefiting patients and public health.

## Declarations

### Availability of Data and Material

Data sharing is not applicable to this article as no datasets were generated or analyzed during the current study.
